# A mixed-methods analysis of moral injury among healthcare workers during the COVID-19 pandemic

**DOI:** 10.1371/journal.pone.0304620

**Published:** 2024-07-03

**Authors:** Arielle A. J. Scoglio, Elisabeth A. Stelson, Iris Becene, Camille Ianne Marquez, Janet W. Rich-Edwards

**Affiliations:** 1 Department of Natural and Applied Sciences, Bentley University, Waltham, MA, United States of America; 2 Department of Epidemiology, Harvard T.H. Chan School of Public Health, Boston, MA, United States of America; 3 Department of Social and Behavioral Sciences, Harvard T.H. Chan School of Public Health, Boston, MA, United States of America; 4 Department of Medicine, Division of Women’s Health, Brigham and Women’s Hospital and Harvard Medical School, Boston, MA, United States of America; St John’s University, UNITED STATES

## Abstract

During the COVID-19 pandemic, healthcare workers faced grave responsibilities amidst rapidly changing policies and material and staffing shortages. Moral injury, psychological distress following events where actions transgress moral beliefs/ expectations, increased among healthcare workers. We used a sequential mixed methods approach to examine workplace and contextual factors related to moral injury early in the pandemic. Using a Total Worker Health® framework, we 1) examined factors associated with moral injury among active healthcare professionals (N = 14,145) surveyed between May-August 2020 and 2) qualitatively analyzed open-ended responses from 95 randomly selected participants who endorsed moral injury on the survey. Compared to inpatient hospital, outpatient (OR = 0.74 [0.65, 0.85]) or school clinic settings (OR = 0.37 [0.18, 0.75]) were associated with lower odds of moral injury; while group care settings increased odds (OR = 1.36 [1.07, 1.74]). Working with COVID+ patients (confirmed+ OR = 1.27 [1.03, 1.55]), PPE inadequacy (OR = 1.54 [1.27, 1.87]), and greater role conflict (OR = 1.57 [1.53, 1.62]) were associated with greater odds of moral injury. Qualitative findings illustrate how outside factors as well as organizational policies and working conditions influenced moral injury. Moral injury experiences affected staff turnover and patient care, potentially producing additional morally injurious effects. Worker- and patient-centered organizational policies are needed to prevent moral injury among healthcare workers. The generalizability of these findings may be limited by our predominantly white and female sample. Further research is indicated to replicate these findings in minoritized samples.

## Introduction

Moral injury can be defined as psychological distress that arises following exposure to a potentially morally injurious event (PMIE), in which an individual acts or fails to act in ways that transgress deeply held moral beliefs and expectations [1,2]. Often, morally injurious events involve betrayal by a person in authority [3]. Moral injury is associated with numerous adverse psychological and behavioral health outcomes, including, posttraumatic stress disorder, depression, anxiety, suicidality, pain, poor sleep and substance use [4–9]. While research on the phenomenon has predominantly focused on military personnel, more recently it has been extended to other workforces, such as nurses working in healthcare settings [10–12]. This line of inquiry is particularly valuable given increasing rates of turnover and attrition within the field of nursing [13,14], anticipated workforce shortages [15], and implications for patient care, outcomes, and healthcare costs.

### Moral injury among healthcare professionals during the COVID-19 pandemic

Prior work examining moral injury centers around military veterans, but the psychological phenomenon has more recently been investigated in healthcare professionals working during the COVID-19 global pandemic [10,12]. Work broadly represents an important social determinant of health, which can yield benefits but also potentially health and social costs [16]. PMIEs within the healthcare sector can involve a variety of high stakes situations, including: caring for COVID-19 patients who are dying without contact with family members, witnessing COVID-19 related unethical healthcare practices and not intervening, and triaging or discharging due to lack of institutional resources COVID-19 patients who need a high level of care [10]. Exposure to PMIEs has been associated with poorer psychosocial functioning, psychological distress, and suicidal ideation [10,17]. Healthcare professionals during the COVID-19 pandemic are especially at risk of being exposed to PMIEs and experiencing moral distress (a precursor to moral injury; [18], which has been previously associated with COVID-19 related posttraumatic stress symptoms, burnout, work stress, and interpersonal difficulties [11]. Certain work-related factors, such as organizational support (the perceived degree to which the organization cares about the worker’s satisfaction, well-being, and contributions), safety standards, and perceived fairness of wage reductions may help ameliorate the negative effects of PMIE exposure [19,20]. It is possible that healthcare workers who regularly interact with COVID-19 patients or work in acute care settings may face higher exposure to PMIEs as a result of acuity and resource rationing within their workplace setting. Working conditions may contribute to worker stress and conflict around their professional role, arising from incongruent expectations around work tasks, role, and evaluations [21]. Literature on moral injury among healthcare workers in this context is limited but growing, with most research focused on a single work context (rather than comparing multiple work settings and conditions) and not necessarily accounting for the lived experience of healthcare workers- as both workers and people with lives outside of the workplace.

### Theoretical framework

**Fig 1:**
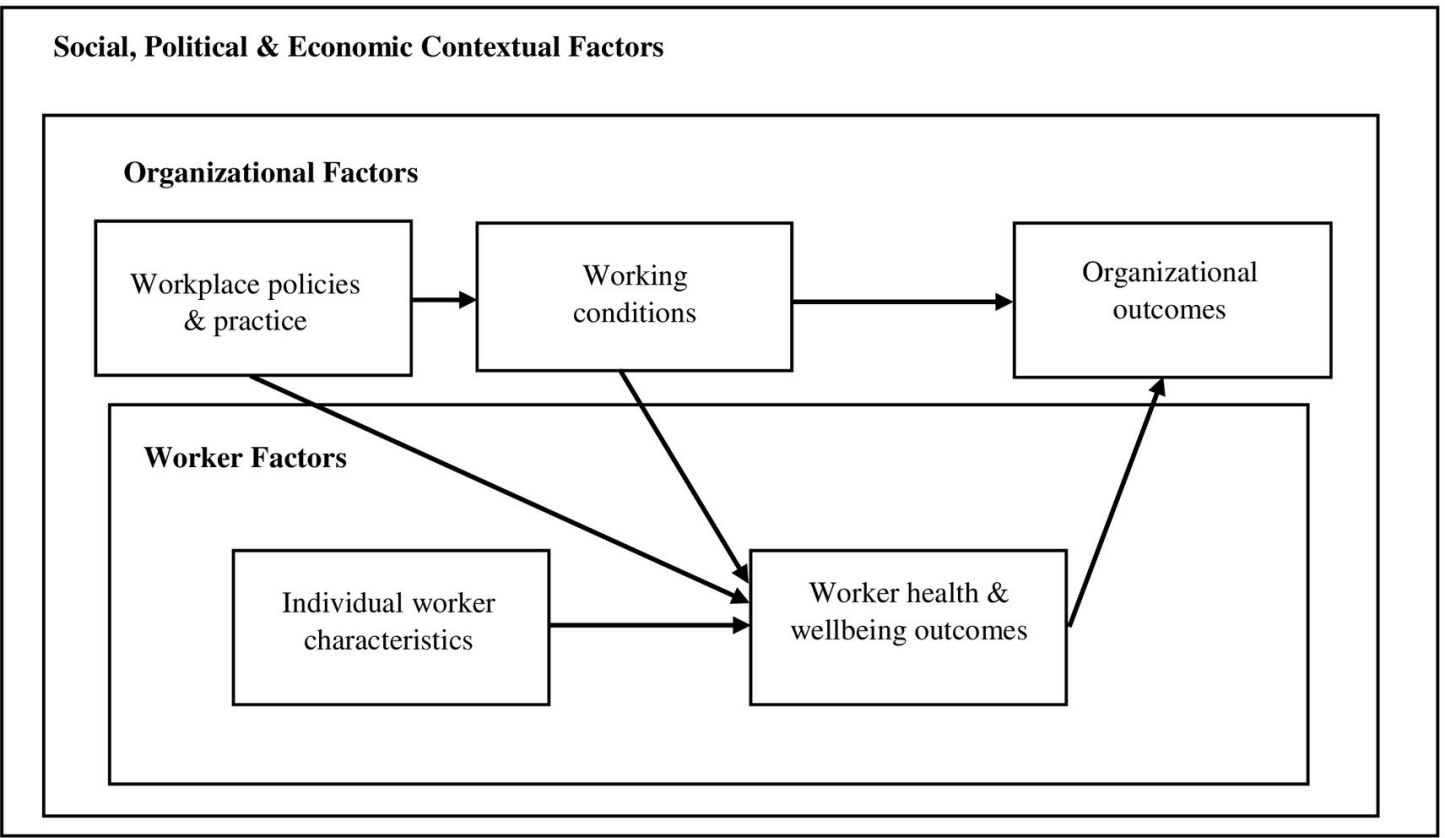
General relationship between Total Worker Health^*®*^ constructs and ecological levels modified from Sorensen et al. (2021) [16] conceptual model.

We applied the Total Worker Health^®^ framework to our analyses. Total Worker Health^®^ is a comprehensive framework for evaluating how contextual factors (such as social, political, and economic policies, events, and environments); worker characteristics and homelife; policies, practices, and programs developed within the organization; and working conditions affect worker health and wellbeing as well as organizational outcomes [17,22]. The ecological levels of this framework, modified from Sorensen et al.’s social contextual model is available in Fig 1 [17].

To date, the phenomenon of moral injury has not been analyzed from a Total Worker Health^®^ perspective, although the framework has been applied to health and social service settings [23,24]. However, this framework is particularly suited to explore contributors to moral injury in healthcare settings given the potential for conflicting priorities between patient, workers, and healthcare organizations. In the context of the pandemic, rapidly changing policies, health care resource allocation needs, and organizational finances within a hierarchical work environment could all be implicated in the moral injury of healthcare workers during this time of collective stress. Analysis from a Total Worker Health^®^ perspective may also help illustrate the potential implications for organizational outcomes, which may include patient outcomes, insurance reimbursements, staff turnover, staffing shortages, and profits and revenue.

### Present study

The present study expands current knowledge about moral injury among healthcare workers during a unique and historic time—the COVID-19 global pandemic. Using a Total Worker Health^®^ theoretical framework as a guide, we utilize both quantitative and qualitative data to examine factors that may contribute to moral injury across multiple ecological levels. Data were analyzed using a sequential mixed methods design [25], where both quantitative and qualitative data were collected at the same time, but analysis of the qualitative data was used to provide context and inform quantitative regression models and their interpretation. Qualitative analyses of open survey responses were exploratory and guided by the following questions: 1) what are the occupational situations in which healthcare workers incur moral injury? 2) what contextual or individual factors contribute to moral injury? and 3) how does moral injury impact healthcare organizations? In our analysis of quantitative survey data, we hypothesized that 1) working conditions which placed workers at greater risk of exposure to COVID-19 (e.g. working with COVID+ patients and not being provided adequate PPE) or 2) created internal conflict about one’s professional role, and 3) settings with more acuity (e.g. inpatient and group care), would be associated with higher moral injury.

## Methods

### Study design and population

The current study consisted of participants from three prospective cohorts: The Nurses’ Health Study II (NHS II), the Nurses’ Health Study 3 (NHS3), and the Growing Up Today Study (GUTS). NHS II is an ongoing cohort study of women, which enrolled 116,429 registered nurses aged 24–42 years residing in 14 US states in 1989. GUTS participants are offspring of NHS II participants and were between 9 and 17 years of age at recruitment in 1996 (N = 27,793), a subset of whom became healthcare workers. NHS3 is a currently recruiting cohort established in 2010 and originally enrolled female registered nurses, licensed practical/vocational nurses, or nursing students born on or after January 1, 1965, who were living in the US or Canada. Between April-August 2020, participants from these three cohorts who had completed the most recent cohort questionnaire were invited to complete an online COVID-19 mixed-method survey designed to examine experiences of participants (at time of survey not all participants worked in healthcare) during the pandemic [26,27]. Following this initial survey, respondents completed a series of follow-up questionnaires with the last quarterly questionnaire completed between March-October 2021. Our analytic sample was restricted to active healthcare workers (defined as professionals working in healthcare facilities where patients are being seen, including home health workers traveling to patient homes to see patients) who had answered the question about moral injury in the Month 1 survey. Although two of the cohorts enrolled registered nurses initially, not all participants continued to work as nurses, and there were several different types of healthcare provider roles represented among active healthcare workers. Our final quantitative analytic sample included 14,516 participants (53.4% (n = 7744) from NHSII, 41.2% (n = 5977) from NHS3, and 5.5% (795) from GUTS). The study protocols were approved by the Institutional Review Boards of the Brigham Women’s Hospital and Harvard Medical School and participant return of questionnaires implied informed consent, but written or verbal consent was not documented or witnessed. Please see S1 Fig for more detail on participant derivation for this study. Authors do not have permission to share data, which is managed by Channing Division of Network Medicine.

Qualitative data were extracted from an unprompted open comment box that appeared at the end of the section of survey questions focused on work (role conflict scale items, moral injury item). We randomly selected 100 participants who endorsed having experienced moral injury on the quantitative scale in the Month 1 survey and made a comment in the comment box to analyze. Five individuals were excluded because the content of their comment was not relevant to the current study (ex. comment included only a period). The qualitative analytic sample included 95 female participants.

### Quantitative measurement

#### Outcome

Moral injury was measured in the Month 1 survey using a single item modified from the Moral Injury Event Scale [28]: “Since March 1, 2020, I am troubled by having acted or failing to act in ways that violated my own morals or values.” Participants rated this item on a 7-point Likert scale (1 = strongly disagree to 7 = strongly agree). Participants were considered to have endorsed experiencing moral injury if they rated a 5, 6, or 7 (somewhat agree, agree, and strongly agree) on this item. We dichotomized this item because we were interested ever experiencing moral injury rather than potential severity.

#### Work-related exposures

In the Month 1 survey, workplace setting (inpatient hospital, outpatient hospital/clinic, group care, school clinic, home health), contact with COVID-19 positive patients (confirmed, presumed, not that I know of, no patient contact), personal protective equipment (PPE) adequacy (defined as adequate, inadequate, not applicable; [26]), and occupational role conflict score. Role conflict was assessed using 4 items modified from the Role Conflict Subscale of the Role Stressor Scale. Participants were asked to rate their level of agreement to statements (e.g., “Since March 1, 2020, I have to bend a rule or policy to carry out my work,” and “Since March 1, 2020, I work on unnecessary things”) using a Likert scale from 1 = strongly disagree to 7 = strongly agree [29]. A sum score was calculated with higher scores indicating greater role conflict, consistent with prior work modifying this measure [30,31]. Cronbach’s alpha for this modified measure was α = 0.74; [32], indicating good internal consistency.

#### Potential confounders

Demographic variables included the following: age (continuous, rescaled by decade for interpretability in tables), sex (binary), racial identity (White, Black, Asian, Hispanic or Other). We included these as potential confounders because they each may be associated with the exposures and moral injury; prior literature has found differences in moral injury development or presentation based on age [33],sex [34], and racial identity [35].

### Quantitative analysis

To address our quantitative aims, we used multivariable logistic regression models with generalized estimating equations (GEE) to estimate the association between identified exposures and sociodemographic characteristics with reported moral injury. GEE is appropriate for non-normal data with binary outcomes and provides robust error variance. We first fitted unadjusted bivariate models and next fitted 3 models to identify the independent contributions of different work-related factors adjusted for age, sex, and racial identity: Model 1 assessed the effect of workplace setting; Model 2 assessed the effect of caring for COVID+ patients; Model 3 assessed the effect of adequate PPE. After qualitatively identifying the centrality of role conflict in PMIEs, we developed a fourth model with a modified role conflict score exposure. Our final fifth model included all four main exposure variables and adjusted for covariates. All analyses were conducted using SAS 9.4 with an α significance level p<0.05.

### Qualitative analysis

Using open coding of a sample of written survey responses (n = 95) and a priori conceptualization of moral injury based upon common definitions in the literature [1,2,6], we developed a code book that contains codes pertaining to 1) descriptions of a potentially morally injurious event (PMIE) and 2) health and interpersonal consequences of PMIE. Codes for exposure to PMIEs included experiencing or witnessing the disturbing actions or inaction of the healthcare professional, healthcare leadership, patients, and people in participants’ larger social environments. Codes for consequences of PMIE exposure included mental health distress symptoms, health behavior changes, and physical health symptoms. All written responses were entered into NVivo12 (QSR International Pty Ltd.). Two trained qualitative researchers independently coded all 95 written records. Inter-rater reliability (IRR) was calculated after the first 25 records were independently double coded; Cohen’s kappa = 0.85 (considered excellent agreement; [36]). Following IRR evaluations, the coders made nominal adjustments to the codebook and continued coding the final 70 records. A third researcher then grouped and summarized data within and between codes to structure an immersion-crystallization approach [37] to identify themes and subthemes through which to understand moral injury experiences and consequences. A Total Worker Health^®^ theoretical framework was employed in this analysis phase to identify how factors both within and outside of the workplace shaped participants’ moral injury experiences [16,22].

## Results

### Quantitative findings

Our full quantitative sample consisted of 14,516 individuals who had complete data on the moral injury question. Most identified as female (98.9%), with a mean (SD) age of 51.7 (12.7) years. Most participants identified as non-Hispanic White (96.1%). 50.0% reported working in an inpatient setting, 83.4% reported adequate PPE, and 9.9% reported experiencing moral injury at work since March 1, 2020. A summary of sociodemographic characteristics and work-related factors in our analytic sample is available in Table 1. Of note, at the final COVID-19 survey (approximately one year later) 42.2% of participants who reported moral injury in Month 1 (N = 1043) had quit or seriously considered quitting their job, compared to 21.0% of those who had not reported moral injury at Month 1.

**Table 1:** Summary of sociodemographic and work-related factors in quantitative and qualitative samples.

	Quantitative Sample (N = 14,516)		Qualitative Sample (N = 95)	
	% (N)	M (SD)	% (N)	M (SD)
Age		51.7 (12.7)		52.9 (11.3)
Sex				
Female	99 (14,358)		100 (95)	
Male	1 (158)		-	
PPE Adequacy				
Adequate	83 (12,105)		80 (76)	
Inadequate	6 (818)		10 (9)	
Not Applicable No response	11 (1,593)		4 (4) 6 (6)	
Workplace Setting				
Inpatient Hospital	50 (7,261)		61 (58)	
Outpatient	39 (5,592)		28 (27)	
Group Care	5 (717)		7 (7)	
Home Health	4 (645)		3 (3)	
School Clinic	2 (301)		-	
Contact with COVID Patients				
Confirmed Cases	7 (1,059)		7 (7)	
Presumed Cases	12 (1,656)		23 (22)	
Not that I know of	74 (10,502)		67 (64)	
No patient contact	7 (1,075)		2 (2)	
Racial Identity				
White	96 (13,955)		98 (93)	
Hispanic	1 (65)		1 (1)	
Black	1 (163)		-	
Asian	2 (230)		1 (1)	
Other	1 (103)		-	
Moral Injury	10 (1,436)		100 (95)	
Role Conflict		3.3 (2.5)		5.8 (2.2)

Note: Means (M) and standard deviations (SD) are provided for continuous variables; numeric frequencies and percentages are provided for categorical variables.

9.9% of participants reported experiencing moral injury. We conducted five multiple regression models. In each of our first four models, we focused on a specific work-related exposure and adjusted for potential confounders: age, racial identity, and sex. Our fifth and final model fully adjusted for all work-related exposures and covariates (see Table 2 for a summary). In our first multiple regression model (Model 1 in Table 2) we examined the relationship between workplace setting and moral injury. Compared to participants working in an inpatient hospital setting (50.0% of the sample), participants working in a group care setting had 41% higher odds of reporting moral injury (OR = 1.41 [95% CI 1.13, 1.76]). Participants working in an outpatient (OR = 0.63 [95% CI 0.56, 0.71]), school clinic (OR = 0.32 [95% CI 0.17, 0.58]) or home health setting (OR = 0.64 [95% CI 0.47, 0.88]) had significantly lower odds of reporting moral injury compared with participants working inpatient. In our second model (Model 2 in Table 2), we examined contact with COVID-19 positive patients as our main exposure. Working with confirmed COVID-19 (OR = 1.76 [95% CI 1.46, 2.11]) and presumed COVID patients (OR = 1.94 [95% CI 1.67, 2.25]), compared to not working with COVID+ patients, was associated with significantly higher odds of moral injury. In our third model (Model 3 in Table 2), we examined reported PPE adequacy. Participants with inadequate PPE were 2.5 times more likely to report moral injury (OR = 2.53 [95% CI 2.11, 3.03]). In Models 1–3 sex and racial identity were not associated with moral injury, but a protective effect was observed for older age; for every decade increase in age, the odds of moral injury decreased 16% (e.g. Model 1 OR = 0.84 [95% CI 0.80, 0.88]). In our fourth model participants with higher role conflict scores were at increased odds of reporting moral injury (OR = 1.60 [95% CI 1.56, 1.65]). In this model participants who identified as Black were at increased odds of reporting moral injury compared to white participants (OR = 1.91 [95% CI 1.16, 3.16]). The wide confidence interval for this odds ratio suggests a lack of precision, likely due to the small number of participants who identified as Black in the sample (1.1%, n = 163). Older age continued to have a protective effect, but it attenuated compared with previous models (OR = 0.91 [95% CI 0.87, 0.95]).

**Table 2:** Summary of cross-sectional moral injury regression model results.

	Model 1: Workplace Setting	Model 2: COVID+ patient contact	Model 3: PPE inadequacy	Model 4: Role Conflict	Model 5: Full Model
	*OR (95% CI)*	*OR (95% CI)*	*OR (95% CI)*	*OR (95% CI)*	*OR (95% CI)*
Age (decade)	0.84 (0.80, 0.88)*	0.85 (0.82, 0.89)*	0.83 (0.80, 0.87)*	0.91 (0.87, 0.95)*	0.93 (0.89, 0.98)*
Sex					
Male (vs. Female)	1.51 (0.88, 2.59)	1.51 (0.88, 2.60)	1.45 (0.84, 2.50)	1.32 (0.72, 2.43)	1.26 (0.68, 2.31)
Racial Identity (vs. White)					
Hispanic	0.38 (0.12, 1.22)	0.37 (0.12, 1.22)	0.34 (0.10, 1.14)	0.39 (0.13, 1.20)	0.37 (0.12, 1.13)
Black	1.50 (0.96, 2.35)	1.47 (0.94, 2.29)	1.41 (0.89, 2.22)	1.91 (1.16, 3.16)*	1.84 (1.10, 3.09)*
Asian	1.29 (0.87, 1.92)	1.27 (0.86, 1.90)	1.28 (0.86, 1.89)	1.25 (0.80, 1.95)	1.27 (0.81, 2.01)
Other	1.50 (0.86, 2.63)	1.47 (0.84, 2.5)	1.50 (0.85, 2.65)	1.35 (0.73, 2.50)	1.28 (0.69, 2.39)
Workplace Setting (vs. Inpatient)					
Outpatient	0.63 (0.56, 0.71)*	-	-	-	0.75 (0.65, 0.86)*
Group Care	1.41 (1.13, 1.76)*	-	-	-	1.37 (1.07, 1.74)*
Home Health	0.64 (0.47, 0.88)*	-	-	-	0.94 (0.67, 1.32)
School Clinic	0.32 (0.17, 0.58)*	-	-	-	0.38 (0.19, 0.76)*
Patient Contact (vs. no COVID+)					
Presumed COVID+	-	1.94 (1.67, 2.25)*	-	-	1.25 (1.06, 1.48)*
Confirmed COVID+	-	1.76 (1.47, 2.12)*	-	-	1.27 (1.04, 1.55)*
PPE (adequate v inadequate)	-	-	2.53 (2.11, 3.03)*	-	1.55 (1.28, 1.88)*
Role Conflict	-	-	-	1.60 (1.56, 1.65)*	1.58 (1.53, 1.62)*

Note: All models adjusted for age (continuous but rescaled by decade for interpretability; decades ranged from 20–80, maximum age 74 years), racial identity and sex as potential confounders. OR = odds ratio; 95% CI = 95% Confidence Interval.

* = p<0.05.

Our final model (Model 5 in Table 2) adjusted for all four work-related exposures, and all four remained significantly associated with moral injury. Odds ratios attenuated for most exposures but remained statistically significant (Group Care Setting OR = 1.37 [1.07, 1.74], Outpatient Setting OR = 0.75 [0.65, 0.86], School Clinic Setting OR = 0.38 [0.19, 0.76], Confirmed COVID+ contact OR = 1.27 [1.04, 1.55], Presumed COVID+ contact OR = 1.25 [1.06, 1.48], PPE inadequacy OR = 1.55 [1.28, 1.88], Role Conflict OR = 1.58 [1.53, 1.62]). Home health setting, as compared to inpatient work setting was no longer associated with less risk of moral injury. Again, in this model we observed increased odds of moral injury among Black-identified participants compared to white counterparts (OR = 1.84 [95% CI 1.10, 3.09]) and a small protective effect of older age (OR = 0.93 [95% CI 0.89, 0.98] per decade).

To further assess the influence of the role conflict score, we conducted a supplementary analysis of individual items from the Role Conflict subscale of the Role Stressor Scale [29]. These items were significantly correlated with moral injury in descriptive analyses (see S1 Table). Endorsement of all four occupational conflict items were significantly associated with moral injury in a fully adjusted model. The results of this model are summarized in S2 Table.

### Qualitative findings

The mean age of respondents in our qualitative sample was comparable to our quantitative sample: 52 years (SD = 11.3, range = 25–70 years). About half were from the NHSII cohort (56.8%; n = 54); 43.2% (n = 41) were from the NHS3 cohort. All identified as female and nearly all identified as non-Hispanic White (97.9%). Qualitative respondents more frequently reported working in an inpatient hospital setting (61.1% vs. 48.8%), worked with more presumed COVID+ patients (23.2% vs. 11.3%), and reported higher occupational role conflict scores (5.8 vs 3.3). Since the qualitative sample was identified based on high moral injury scores and these factors were related to moral injury in qualitative analysis, the high proportions observed in this subsample is expected.

Throughout written survey responses, participants with high moral injury quantitative scores described a variety of contributors to their experiences of work-related moral injury during the COVID-19 pandemic. Some of these contributors occurred externally to the workplace while others originated within the workplace. These contributors have been organized according to Total Worker Health^®^ constructs (socio-political contextual factors, workers’ life outside of work, workplace policies and practices, and working conditions) in Table 3 and evidenced with illustrative quotes.

**Table 3:** Mapping total worker health^*®*^ constructs to moral injury themes.

Total Worker Health Construct		Theme	Illustrative Quote
External to the organization	Socio-political contextual factors	Confusion over federal COVID-19 policies	It’s hard to be a nurse using the best evidence-based practices when there is no evidence to support our practices.
		Distrust of federal leadership	Lost a large amount of trust from…our national organizations (CDC) for the inconsistencies in recommendations and basically advising us (or making it seem like it’s okay) to now suddenly do things that were 100% not recommend in the past (i.e. wearing surgical masks or other PPE for multiple uses and patients for 2 weeks straight).
		Disregard for public health guidelines	General public doesn’t understand or respect science, even as my community is realizing a second wave of COVID cases.
		Combatting misinformation	Seeing people believe this is a hoax is a slap in the face!
	Workers’ life outside of work	Fear of exposing loved ones to COVID	Now, I feel guilty and worried that I might have brought it home to them [family].
		Conflicting obligations (protect family vs. care for patients).	I now work 6–7 days per week and taking a day off seems selfish and causing me stress so I avoid taking days off.
Internal to the organization	Workplace policies & practices	Leadership focused on cost-saving at expense of worker and patient wellbeing	Ancillary staff (support staff) are not being brought back from furlough until the end of the fiscal year to make up for lost revenue.
		New policies about essential care did not match expectations for ethical care	The cases are crucial with many cancer diagnosis, urinary stents in for long periods of time making patients risk spread of cancer or infections.
		Patient visitation restrictions cruel	It is traumatizing to separate moms and babies.
		Policies issued without implementation plans made it hard to maintain care	Currently feel like hospital standards have become so eroded as to not have a standard of care except in name/writing only!
		Hierarchical and inaccurate communication from administration	The manager feels that she is the filter for information for the staff and it is not shared in a timely manner or the information/processes are incomplete.
		Policies put in place do not recognize the need to support workers	The lack of interest from upper management in taking care of nurses and caring about what they have to say. I’m at a small hospital. One 24-bed unit was prescribed to be the "Covid unit." With all 24 beds being isolation for Covid, there was no change to the staffing grid for that floor, and no planned extra personnel for the floor.
		Fear of retribution if staff voiced opinions or complaints	If reporting ’bullying’ [I] will jeopardize my employment—and using the union—so I go day to day.
		Inconsistent PPE policies/ enforcement placed patients and workers at risk	The enforcement of wearing PPE is lax, to say the least. The staff wears masks "only if they want to," whether they are surgical masks or cloth masks.
	Working conditions	Staffing shortages made staff feel unsupported and inhibited quality care	Staffing is poor and our volumes have increased without needed support to manage the volume.
		Lack of training on new policies made staff feel unprepared	I was required to sign an affidavit stating I was trained and competent. I was very angry. Often everything I do is for the first time and I have no one to show me how.
		Witnessing or experiencing differential staff treatment and discrimination	The employer’s policies on anyone with a disability cannot work and must file for a leave of absence. The hospital/corporation indicated there were unable to accommodate any disability and that my choices were to get a physician to write a note that I didn’t need any restrictions for life-long disability or I would be on unpaid leave of absence [LOA]. And they still needed me to work on my LOA …but not tell anyone about it …We need a workforce that embraces all types of abled peoples and does not discriminate against anyone with a disability using the facade of safety, health, or other pretense that garners corporations strategy for ousting specific groups of people, like those with disabilities.
		Unsupportive supervision	My supervisor doesn’t seem to care.
		Availability of PPE to keep workers safe	Reprocessed masks are never correctly returned to the same user and they are reprocessed infinitely. The provision of N95 in my O.R. department DOES NOT MEET any of the FDA guidelines for the decontamination and utilization of reprocessed N95.
		Use of PPE and infection policies inhibits providing the best care to patients	I have a lot of moral distress with the concept of protected code blue. Patient in cardiac arrest and we delay CPR to don PPE.
		Unrealistic expectations of workers amidst challenging conditions over which workers have little control	It was left to frontline ICU managers and others to figure out how to operationalize this [COVID response]. Which we did, but had SOOO much else to do, and did not have the authority or power or influence to get what we needed from other departments without senior leadership influence. Terribly frustrating, while each of us has at least 60 direct reports, staff who are working directly with covid patients, anxious, have family issues, medical issues, need to take time off, etc …

Many participants described how social and political contextual factors originating outside of the workplace interfaced with personal work experiences or sense of professional self to contribute to moral injury. Describing their efforts to combat COVID-19 at work while simultaneously witnessing nonchalance among the public, one participant wrote: “It’s a moral struggle to be trying to prevent illness at work and then sense all that work being undone when few people are wearing masks or distancing in the stores, and food service workers aren’t wearing masks when we pick up food.” Another participant noted the distress they experienced when an elected official contradicted public health experts: “I feel very stressed when our president does not defer to the educated experienced professionals who should be at the forefront of this pandemic.”

Participants also described how their homelife interacted with work responsibilities to contribute to internal conflict, conflicting demands, and moral injury. A number of participants described fears of accidentally exposing a loved-one to the virus as a result of their caring for COVID-19-positive patients at work. As one participant stated: “I deal with risking possible exposure and then coming home to my mom who is a high-risk individual.” Others described a sense of conflicting responsibility as a healthcare provider during a public health crisis and as a person with family responsibilities. One participant explained this internal conflict: “When I spoke of feeling the need to volunteer, they [children] were distraught, so I chose my children’s well-being over my guilt.”

Within the workplace, conflict between new COVID-related policies and practices, implementation and communication of these policies and practices, and prioritization of institutional finances over patient and worker health and wellbeing produced a variety of PMIEs for participants. Eight workplace policy and practice themes that contributed to participants’ experience of moral injury emerged in analyses (Table 3). Some participants reacted to what they perceived as poor decision-making by leadership, which appeared to prioritize financial gain over or patient and worker safety (e.g. “I have had unnecessary patient exposure due to financial interests of the hospital.”). Other participants wrestled with enforcing effective COVID-19 containment policies at the expense of their patients’ emotional wellbeing. For example, one participant shared: “It is necessary but terrible that we cannot have visitors with patients, that patients are suffering and dying without them.” Participants described feelings of distrust, neglect, and abandonment by organizational leaders during the pandemic and limited support for workers permeated many of workplace policy and practice themes. Participants often described how their employers minimized their needs as workers, reportedly “lied at times about the safety of [their] workplace,” or felt that leadership did not care about the emotional and physical hardship they experienced “beyond getting photo-ops and a happy blurb in the news about how amazing they’re being for their staff.” Several participants explicitly described fear of retaliation by leadership if they were to advocate for the health and safety of patients and workers. As one participant disclosed: “I almost filed an OSHA complaint, but I worry about the repercussions.”

Both material and psychosocial working conditions surfaced as contributors to morally injurious experiences. Numerous participants described staffing shortages driven by hiring and financial decisions of their organizations (not workforce shortages) and how this affected their ability to provide care. One participant succinctly stated: “They will not hire enough nurses to care for patients. They have actual beds.” Participants also noted that limited availability of clean and high-quality PPE affected their sense of safety. Similar to the workplace policy and practice themes, participants also reported feeling conflicted about how the implementation of COVID-19 safety precautions were often at odds with responding to patient needs. As one participant wrote: “I am forced to try to do psych assessments while at the med window. No quality care at all.” Participants also identified a variety of psychosocial working conditions that contributed to their moral injury, particularly witnessing or experiencing discrimination and unequal treatment of staff (e.g. “I saw first-hand also that physicians, nurses, technologists, housekeeping were treated differently.”); bullying by supervisors (e.g. “Our current and fairly new nursing supervisor is ex-army, a bully, who has threatened the nurses, and never lifts a finger to help out, even in an emergency or when short a nurse.”); and unrealistic expectations by superiors (e.g. “My level of burnout is more from lack of support and realistic expectation of my supervisor.”) Many of these working conditions can be described as PMIEs.

A Total Worker Health^*®*^ approach postulates that contextual factors, worker characteristics, worker health and well-being, workplace policy and practices, and working conditions coalesce to influence organizational outcomes, such as worker engagement, staffing turnover, and patient care (Fig 1). Analysis of participant responses yielded four themes indicating the impact of moral injury on worker engagement and staff turnover: 1) demoralization of workers who could not meet their own patient care standards; 2) emotional and physical exhaustion of workers contributed to an inability to complete tasks or be fully engaged; 3) workers choosing to leave their current jobs; and 4) workers choosing to leave direct patient care or healthcare workforce. Participants also described additional moral injury and emotional distress from witnessing or participating in what they considered to be poor or unethical patient care. That is, patient-related organizational outcomes seemed to contribute to additional moral injury. For example, one participant disclosed how they experienced “a lot of emotional distress relating to inequities in care that were deepened or laid bare by changes.” Three primary themes surfaced through analysis of patient care and outcomes statements: 1) perceived inability to intervene when witnessing poor care; 2) witnessing or participating in avoidable patient death; and 3) witnessing inequities in patient care. Additional illustrative quotes for each of these themes can be found in Table 4.

**Table 4:** Bidirectional influence of organizational outcome themes and moral injury.

Impact on organizational outcomes	Organizational outcome theme	Illustrative quote
Worker engagement and turnover	Demoralization of workers who could not meet their own patient care standards	I can’t even tell you how many times in the last week I have heard our nurses say, "My heart hurts," knowing that in the past we were able to do so much better for these people.
	Worker exhaustion contributed to inability to complete tasks or be engaged.	I hit a critical burnout level and completely fell apart this week.
	Anger and frustration directed at healthcare workers who are not addressing COVID.	I am extremely irritated, often angry, at nurses who have shunned working with COVID-positive patients or continue to fight to keep visitors out of facilities.
	Workers left direct patient care/ healthcare	I am looking for a new job. I want out of direct patient care.
Patient care & outcomes	Workers felt unable to intervene in poor care	Stress over witnessing a supervisor do something unethical but unable to do much about it.
	Avoidable patient death	Having had transplants halted at our center and watch our patients die is unacceptable.
	Inequities in patient care	I compare his outcome to that of another patient who was actually much more ill but recovered drastically over time. The first patient was black, the second patient was white. The family of the first patient deferred to the expertise of the medical team. I wonder how different the outcome of the first patient would have been if his family had behaved the way the white family did.

## Discussion

Our mixed-methods sequential analysis yields important insight into the role of both factors external to the workplace, such as the larger socio-political environment and workers’ home lives, as well as factors that are internal to organizations, such as workplace policies and working conditions, and how these may have contributed to moral injury among healthcare workers early in the COVID-19 pandemic. Our findings further highlight the responsibility healthcare organizational leaders have to protect workers from exposure to PMIEs in their working environment. Our quantitative results suggest the type of health care setting (which then shapes the policies, practices, and working conditions within) mattered for moral injury, with inpatient and group care settings increasing likelihood of reporting moral injury. Similarly, inadequate PPE was strongly associated with moral injury in quantitative models and came up in multiple qualitative themes where participants worried about their own safety, the safety of patients, and the safety of loved ones at home who they might inadvertently expose.

Across qualitative themes, the role of internal, interpersonal, and organizational conflict consistently emerged. Participants wrote about rapidly changing policies and practices that seemed differentially implemented, potentially putting them at risk for contracting COVID while fearing retribution for voicing complaints. Interpersonally, participants described bullying and unsupportive supervision as well as witnessing unethical or discriminatory conduct by other workers. This aspect of betrayal by an institution or authority figures is consistent with prior research distinguishing moral injury from other posttraumatic sequalae [3] In recognition of the central role of workplace conflict in moral injury from our qualitative findings, we incorporated role conflict into our quantitative analyses. Role conflict remained a strong risk factor for moral injury even when adjusting for other working conditions (e.g. inadequate PPE and contact with COVID patients) and high acuity settings (group care and inpatient). The odds ratio for working with COVID-positive and presumed positive patients attenuated (reducing from 1.92 to 1.25) in the fully adjusted model, suggesting that inadequate protective resources and workplace conflict—likely operating at intrapersonal, interpersonal, and organizational levels—may contribute to moral injury more substantially than increased exposure via COVID patient contact. In the work conflict and fully adjusted models, Black identified participants had nearly 2 times higher odds of reporting moral injury compared to white identified workers. We want to acknowledge that race is socially constructed and may be conceptualized as a proxy for racism [38]. Given the discriminatory care witnessed by participants and reported differential treatment across job roles (which are socially patterned), the impact of experiencing such events may be particularly damaging to healthcare workers identifying as Black who already face additional systemic challenges outside of PMIE exposure. We do want to note that the small number of participants who identified as Black in our sample contributed to wide confidence intervals in this analysis, and more racially diverse healthcare workforce samples would help illuminate the relationship between racial discrimination and moral injury.

Workers in this study also wrestled with deep internal dilemmas of providing care they believed was not best for the patient but felt necessary to protect themselves and others from COVID-19. Interestingly, this reveals a two-sided problem. On one side, enforcing policies to protect workers (i.e. PPE) may protect against moral injury since workers may feel valued and protected by their institution. Indeed, workers with inadequate PPE were at much higher odds of experiencing moral injury, even when accounting for other workplace contributors. At the same time, such policies could contribute to moral injury by reducing patient-centered care and inadequately responding to patient needs (i.e. workers donning PPE before attempting resuscitation of a patient or conducting patient interviews through plexiglass barriers). From an organizational perspective, some PMIE exposure in a pandemic context may be necessary (PPE or restricted visitation policies), while others are avoidable (e.g. unsuportive supervision, discriminatory care, poor communication).

**Fig 2:**
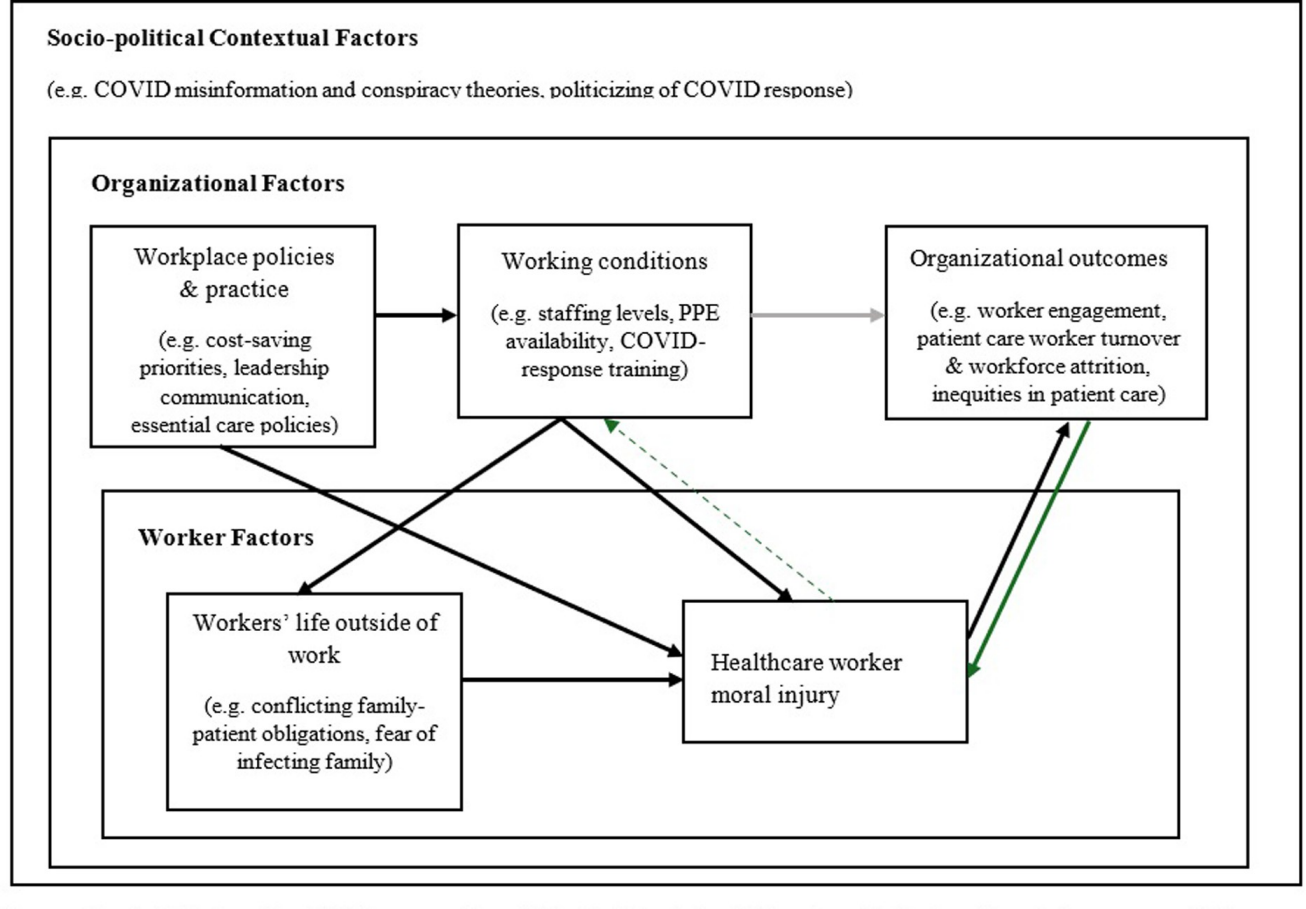
Situating healthcare worker moral injury within a Total Worker Health^*®*^ framework. General Total Worker Health^®^ framework modified to illustrate bidirectional relationships between moral injury, working conditions, and organizational outcomes as well as feedback loops by which some organizational outcomes contribute to additional moral injury and shape working conditions. (Grey arrows denote feedback loop, dotted arrow denotes theorized relationship).

While our quantitative findings identify specific factors that may contribute to moral injury (e.g. inpatient and group care settings, inadequate PPE), our qualitative analysis provides nuanced context about these working conditions and how the relationship between them, moral injury, and organizational outcomes may be bi-directional. With a Total Worker Health^*®*^ theoretical framing, worker moral injury would be conceived of as a form of worker health and wellbeing, contributing to “downstream” organizational outcomes, such as worker engagement and turnover. Analysis of participants written responses, however, indicates that in some situations, moral injury was also the *result* of such organizational outcomes, specifically inadequate patient care and staffing. Participants often described being “emotionally drained” or “falling apart” as a result of their experiences at work during the COVID-19 pandemic—particularly PMIEs involving inadequate patient care. Others described looking for new jobs in healthcare or plans to transition out of the healthcare workforce altogether. These worker outcomes would likely have additional effects on availability and quality of care either through worker engagement or workforce shortages. In this way, organizational outcomes in the theoretical model may have a bidirectional relationship with moral injury, such that working conditions and inadequate care may contribute to worker moral injury, which may further contribute to negative working conditions and patient care. Fig 2 visualizes these conceptual bidirectional influences and feedback loops. It is also important to note that 42% of participants who endorsed moral injury in our study, quit or seriously considered quitting their jobs by the final wave of the substudy (approximately 11 months later). Although outside of the timespan of our analysis, this context is important to consider in light of healthcare workforce shortages [39] and implications for patient care. Taken together with our study findings, it emphasizes the critical need for healthcare organizations to develop and maintain work environments that support their workers and allow them to do their jobs ethically and consistently, even in times of crisis.

### Implications for future research and practice

Our qualitative findings provide an important reminder that healthcare workers have personal lives that cannot be divorced from their professional selves. While perhaps more pronounced during a global pandemic, healthcare is influenced by local and national social, political, and economic factors [40,41]. Our findings emphasize the importance of accounting for these extra-organizational factors when seeking to understand how moral injury—which would not have been observable using the available quantitative data by itself. Healthcare organizations must consider their workers as whole people and understand how personal lives intersect with work. It is in employers’ best interest to be concerned about and take steps to prevent moral injury among workers because it negatively impacts their organizational outcomes, including potentially reducing their workforce.

### Strengths and limitations

The sequential mixed-method design of this study is a strength, as it brings together data from a large, well characterized cohort of healthcare workers into dialogue with a subset of these participants’ experiences shared in their own words during a critical period—the early months of the COVID-19 pandemic. Analyzed from a Total Worker Health^®^ perspective, our qualitative contextualization illustrated the interplay between factors external to the workplace and internal organizational factors on moral injury and also helped to inform and refine quantitative analytic models to investigate the specific contribution of role conflict. These data come from the beginning of the COVID-19 global pandemic, which provides insight into healthcare workers’ experiences at the onset of a public health crisis.

Our study also has several limitations to consider. While our sample was large, it was predominately white and female, and may not generalize to the experiences of all healthcare workers. Similarly, prior literature has revealed sex differences in moral injury and so our findings may not generalize to male healthcare workers [34]. Our quantitative analyses were cross-sectional, and so we were not able to assess the temporality of relationships between factors associated with moral injury. The unprompted comment box from which qualitative data were extracted appeared immediately following the quantitative survey questions about work conflict and moral injury. As we were interested in factors contributing to moral injury, we examined comments made by participants who had reported moral injury on the survey. However, this limits our insight into factors that may have been supportive or protective against moral injury at work. Additionally, analyzing qualitative comments from an unprompted comment box likely yielded different responses than more structured qualitative inquiries would, such as interviews or focus groups on these topics. Future work should consider comparing perspectives of workers with and without moral injury to identify potentially protective factors.

## Conclusion

Our findings suggest that moral injury was experienced by healthcare workers early in the pandemic and that workplace setting, policies, practices, and conditions contributed to moral injury experiences. Qualitative data also revealed that moral injury was tied to organizational outcomes, like staff turnover and patient care, which in turn may produce PMIEs. To protect healthcare workers and maintain high quality and ethical patient care, worker and patient-centered healthcare organizational polices must be implemented effectively, even in times of crisis.

## Supporting information

S1 Fig Participant derivation summary. (DOCX)

S1 Table Workplace conflict item endorsement and correlation with moral injury. (DOCX)

S2 Table Cross-sectional associations with moral injury including individual role stressor scale items. (DOCX)
