# Supplementary material for: A mixed-methods analysis of moral injury among healthcare workers during the COVID-19 pandemic
Source: PLoS One. 2024 Jul 3;19(7):e0304620. doi: 10.1371/journal.pone.0304620 (PMC11221684; doi:10.1371/journal.pone.0304620)
Supplement: S1 Fig — (DOCX) [file pone.0304620.s001.docx]

**Supplemental Figure 1:** Participant Derivation Summary

NHSII

116,429

14,516 active healthcare workers with moral injury month 1 data

NH3

35,852

GUTS

27,793

39,564 (71%) responded

(All female)

Excluded participants who are not active healthcare workers (N=36,239)

55,925 invited to COVID-19 substudy

(All female)

- 5844 died
- 4868 no longer in cohort
- 26610 no email
- 585 paper questionnaires only
- 21076 main cohort questionnaire pending
- 369 opted out of substudies
- 1104 currently in another substudy
- 48 self report dementia
- 40 no longer in cohort
- 2135 no email
- 3034 completed <2 main questionnaires
- 146 died
- 1633 no longer in cohort
- 6920 no email

30,643 invited to COVID-19 substudy

(30,213 female; 430 male)

19,094 invited to COVID-19 substudy

(11,466 female; 7628 male)

12,317 (40%) responded

(11,976 female; 199 male)

6,725 (35%) responded

(4,681 female; 1,827 male)

35,852 with Month 1 data

9,727 with Month 1 data

5,176 with Month 1 data

7,744 respondents (53.4%)

5,977 respondents (41.2%)

795 respondents (5.5%)
