# Supplementary material for: A mixed-methods analysis of moral injury among healthcare workers during the COVID-19 pandemic
Source: PLoS One. 2024 Jul 3;19(7):e0304620. doi: 10.1371/journal.pone.0304620 (PMC11221684; doi:10.1371/journal.pone.0304620)
Supplement: S1 Table — (DOCX) [file pone.0304620.s002.docx]

| Supplemental Table 1. Workplace Conflict Item Endorsement and Correlation with Moral Injury | | |
| --- | --- | --- |
|  | **% (N)** | Correlation with moral injury |
| I work on unnecessary things |  | 0.21 (<.0001) |
| Agreed | 22.6 (2944) |  |
| Disagreed | 65.8 (8565) |  |
| Neutral | 11.6 (1511) |  |
| I am not sure what is expected of me at work |  | 0.24 (<.0001) |
| Agreed | 19.3 (2513) |  |
| Disagreed | 72.5 (9445) |  |
| Neutral | 8.2 (1067) |  |
| I receive incompatible requests from two or more people or groups at work |  | 0.27 (<.0001) |
| Agreed | 29.2 (3795) |  |
| Disagreed | 57.5 (7489) |  |
| Neutral | 13.3 (1737) |  |
| I have to do things that should be done differently at work |  | 0.18 (<.0001) |
| Agreed | 60.3 (7855) |  |
| Disagreed | 26.4 (3439) |  |
| Neutral | 13.3 (1729) |  |
| I have to bend a rule or policy to carry out my work |  | 0.29 (<.0001) |
| Agreed | 29.2 (3808) |  |
| Disagreed | 58.0 (7556) |  |
| Neutral | 12.8 (1669) |  |
| Note: Correlations refer to Pearson’s correlation coefficients. 4 items come from the Role Conflict Subscale and 1 item comes from the Role Ambiguity Subscale of the Role Stressor Scale (Rizzo et al., 1970). Alpha significance level set at *p*<0.05 | | |
