# Supplementary material for: A mixed-methods analysis of moral injury among healthcare workers during the COVID-19 pandemic
Source: PLoS One. 2024 Jul 3;19(7):e0304620. doi: 10.1371/journal.pone.0304620 (PMC11221684; doi:10.1371/journal.pone.0304620)
Supplement: S2 Table — (DOCX) [file pone.0304620.s003.docx]

| Supplemental Table 2. Cross-sectional associations with Moral Injury including Individual Role Stressor Scale Items | | | |
| --- | --- | --- | --- |
|  | ß | 95% CI | *p* |
| Inadequate PPE | 0.32 | 0.12, 0.52 | 0.002 |
| Workplace Setting |  |  |  |
| Outpatient (reference) | - | - | - |
| Group Care | 0.55 | 0.29, 0.81 | <.0001 |
| Home Health | 0.20 | -0.15, 0.55 | 0.271 |
| Inpatient Hospital | 0.26 | 0.12, 0.40 | 0.0003 |
| School Clinic | -0.72 | -1.44, -0.01 | 0.048 |
| No COVID+ patient contact (reference) | - | - | - |
| Worked with confirmed COVID-19 cases | 0.14 | -0.07, 0.35 | 0.194 |
| Work with presumed COVID-19 cases | 0.15 | -0.02, 0.32 | 0.084 |
| Posttraumatic Stress Symptoms | 0.37 | 0.29, 0.45 | <.0001 |
| Racial Identity |  |  |  |
| White (reference) | -- | -- | -- |
| Hispanic | -1.08 | -2.21, 0.05 | 0.061 |
| Black | 0.72 | 0.21, 1.24 | 0.006 |
| Asian | 0.37 | -0.07, 0.81 | 0.099 |
| Female Sex | 0.22 | -0.41, 0.84 | 0.497 |
| Age | -0.01 | -0.01, -0.00 | 0.005 |
| I am not sure what is expected of me at work |  |  |  |
| Agreed | 0.58 | 0.43, 0.72 | <.0001 |
| Disagreed (reference) | -- | -- | -- |
| Neutral | -0.11 | -0.33, 0.12 | 0.352 |
| I receive incompatible requests from two or more people or groups at work |  |  |  |
| Agreed | 0.68 | 0.52, 0.85 | <.0001 |
| Disagreed (reference) | -- | -- | -- |
| Neutral | 0.10 | -0.12, 0.33 | 0.357 |
| I have to do things that should be done differently at work |  |  |  |
| Agreed | 0.64 | 0.40, 0.89 | <.0001 |
| Disagreed (reference) | -- | -- | -- |
| Neutral | 0.36 | 0.05, 0.68 | 0.024 |
| I have to bend a rule or policy to carry out my work |  |  |  |
| Agreed | 1.14 | 0.97, 1.30 | <.0001 |
| Disagreed (reference) | -- | -- | -- |
| Neutral | 0.33 | 0.10, 0.56 | 0.006 |
| Note: 4 items come from the Role Conflict Subscale of the Role Stressor Scale (Rizzo et al., 1970). | | | |
